# Endovascular coiling versus neurosurgical clipping in patients with unruptured intracranial aneurysm: a systematic review

**DOI:** 10.1186/1471-2377-12-99

**Published:** 2012-09-22

**Authors:** Jin Seub Hwang, Min Kyung Hyun, Hyun Joo Lee, Ji Eun Choi, Jong Hee Kim, Na Rae Lee, Jin-Won Kwon, EnJu Lee

**Affiliations:** 1 National Evidence-based Healthcare Collaborating Agency, Changkyung B/D 8 F, 28-7 Wonnam-dong, Seoul, Jongno-gu, 110-450, Korea; 2 Kyungpook National University, 80 Daehakro, Daegu, Bukgou, 702-701, Korea

**Keywords:** Unruptured intracranial aneurysm, Endovascular coiling, Neurosurgical clipping

## Abstract

**Background:**

To compare the effects of endovascular coiling and neurosurgical clipping in patients with unruptured intracranial aneurysm.

**Methods:**

Sixteen electronic databases were searched for articles published between 1950 and July 2010 to compare clinical outcomes of clipping and coiling. Researchers reviewed all searched articles and extracted data independently. The quality of studies and evidence were evaluated using MINORS and GRADEprofiler, respectively. The odds ratio (OR) was calculated using the inverse variance meta-analysis method for each study outcome. To assess heterogeneity of ORs across cohorts, Cochran’s Q statistic and I^2^ were used.

**Results:**

Of 4160 studies, 24 were identified (*n*  =  31865). Clipping resulted in significantly higher disability using the Glasgow Outcome Scale (OR, 2.38; 95% CI, 1.33–4.26) and Modified Rankin Scale (OR, 2.83; 95% CI, 1.42–5.63) when compared with coiling. ORs for complications were also higher with clipping (ORs for neurological and cardiac complications were 1.94 with a 95% confidence interval [CI] of 1.09–3.47 and 2.51 with a 95% CI of 1.15–5.50). Clipping resulted in significantly greater disability in the short term (≤6 m)(OR on the Glasgow Outcome Scale, 2.72; 95% CI, 1.16–6.34), but not in the long term (>6 m)(OR for Glasgow Outcome Scale, 2.12; 95% CI, 0.93–4.84).

**Conclusions:**

Coiling was a better procedure for treatment of unruptured intracranial aneurysm in terms of disability, complications, especially in the short term. Because of the limitations of the reviewed studies, further studies are required to support the present results.

## Background

Recent developments in non-invasive imaging detection, such as magnetic resonance (MR) angiography and computed tomography (CT) angiography, have made screening for unruptured intracranial aneurysm (UIA) possible in the general population [1]. Despite study results showing that it is not cost-effective to screen for asymptomatic UIA [2], such screening has become widespread, and UIA is being identified with increasing frequency.

Patients with UIA always have risks of rupture with intracranial aneurysms. A nationwide study in Sweden showed that the 5-, 10- and 15-year risks of death after subarachnoid haemorrhage (SAH) were 12.9%, 23.6% and 35.4%, respectively [3]. Thus, patients with UIA are often treated to prevent SAH, and these prophylactic treatments include neurosurgical clipping and endovascular coiling. Neurosurgical clipping of the aneurysm was the standard treatment prior to the introduction of detachable coils, but endovascular coiling has recently been increasing [4]. Neurosurgical clipping is recommended over endovascular coiling for young patients and for small or anterior circulation aneurysms, i.e., low-risk cases [5]. Neurosurgical clipping is an order of magnitude more durable than endovascular coiling, but endovascular coiling is less invasive than neurosurgical clipping; thus, it may be suitable for high-risk UIA patients (elderly or posterior aneurysms, etc.) [5].

Using the national inpatient sample database from 2002 to 2008, Smith et al. [6] showed that the majority of ruptured and unruptured aneurysms in the US are coiled. However, which treatment is better remains controversial, and there have been no systematic reviews of patients with UIA. Therefore, an extensive systematic review and meta-analysis were performed to compare the benefits and risks in UIA patients treated with endovascular coiling vs. neurosurgical clipping.

## Methods

### Literature search

We developed and adhered to a protocol for population, intervention, comparison and outcome (PICO) search methods, data extraction, quality assessment, meta-analysis and grading of the quality of the evidence for this systematic review.

Articles Published before July 2010 in 16 electronic databases were searched. These databases included three international databases, ten Korean domestic databases and three Japanese domestic databases: (1) Ovid-Medline, (2) Ovid-Embase, (3) Cochrane Library, (4) KISS ( http://kiss.kstudy.com), (5) KMBASE ( http://kmbase.medric.or.kr), (6) KoreaMed ( http://www.koreamed.org), (7) NDSL ( http://www.ndsl.kr), (8) KiSTi ( http://society.kisti. re.kr), (9) J Cerebrovasc Surg ( http://jkcvs.ksevs.org), (10) J Korean Soc Radiol ( http://www.radiology.or.kr), (11) J Korean Neurosurg Soc ( http://jkns.or.kr), (12) Neurointervention ( http://www.ksin.or.kr), (13) Korean J Stroke ( http://www.stroke.or.kr), (14) JAMAS ( http://www.jamas.or.jp), (15) Medical online ( http://www.meteo-intergate.com) and (16) J-stage ( http://www.jstage.jst.go.jp). Various combinations of Mesh headings and keywords were used, such as “intracranial aneurysm”, “subarachnoid haemorrhage”, “embolisation”, “neurosurgical procedures”, “neurosurgery”, “unruptured”, “coil”, “Guglielmi”, “GDC”, and “clip”. The search was limited to human studies without language restrictions.

### Study selection, data extraction and quality assessment

Eligible studies were those that included adult (≥18 years) patients with UIA. Patients with mycotic, infectious, dissecting or fusiform aneurysms, arteriovenous malformation or arteriovenous fistula were excluded. Case series, case reports and publications that had not undergone peer review were excluded. In addition, studies were required to have directly comparable outcome measures for endovascular coiling and neurosurgical clipping, such as overall death, in-hospital mortality, disability and complications. Disability was regarded as a score of 1–5 on the Modified Rankin Scale [mRS] and a score of 2–4 on the Glasgow Outcome Scale [GOS]. The mRS, a clinician-reported measure of global disability, is widely applied to evaluate stroke patient outcomes, and scores run from no symptoms at all (score 0) to death (score 6) [7]. The GOS assesses outcome after severe brain damage and runs from death (score 1) to good recovery (score 5) [8]. There were various complications, including bleeding or haematoma in the brain, ischemia or infarction, cerebral vessel damage, cerebrospinal fluid fistula, infection, cranial nerve disorder, cognitive impairment, encephalitis, meningitis, embolism, cardiac disease and pulmonary disease.

Four authors (HJL, JEC, JSH and MKH) independently reviewed all searched articles and extracted data using pre-made extraction forms that included study design, follow-up period, inclusion/exclusion criteria, sample size, sex, age, aneurysm location, aneurysm size, race, baseline characteristics, treatment protocol, outcome variables and complications. Disability was calculated based on the percentages of categories other than death/good recovery in mRS/GOS. The numbers of events for outcomes were extracted according to the intention-to-treat principle.

Six authors (HJL, JEC, JHK, JSH, LRL and MKH) independently evaluated the quality of the studies using the Methodological Index for Nonrandomised Studies (MINORS) [9]. In the case of disagreement, consensus was reached through discussion and negotiations with partners. If a consensus could not be achieved within the group, a third party was involved, and then an agreement was reached by majority rule.

### Meta-analysis and grading the quality of the evidence

A meta-analysis was performed to synthesise the outcomes, except for missing data among 24 articles. Binary outcomes were expressed as odds ratios (ORs). The reporting methods for in-hospital mortality were diverse, including the number of patients, unadjusted ORs and adjusted ORs. Therefore, lnOR and standard error were calculated using statistical equations.

Fixed and random effects inverse-variance meta-analysis was used to combine the studies and obtain the average effects and 95% confidence intervals (CI). The subgroup analyses were performed by outcome measurement times (short time, ≤6 months; long time, >6 months) according to medical experts’ opinions.

To assess heterogeneity across studies, funnel plots were visually examined, and Cochran’s Q statistic and the I^2^ statistic were used. Publication bias was also assessed using funnel plots, Begg and Mazumdar’s rank correlation (Begg’s test) and Egger’s linear regression asymmetry test of the intercept (Egger’s test). Finally, the quality of the body of evidence was graded as “high”, “moderate”, “low”, or “very low” [10].

Meta-analyses were conducted using Comprehensive Meta-analysis 2.0 (Biostat, Englewood, NJ, USA), and the quality of evidence and strength of recommendation were graded using GRADEprofiler 3.2.2 (GRADE Working Group).

## Results

**Figure 1:**
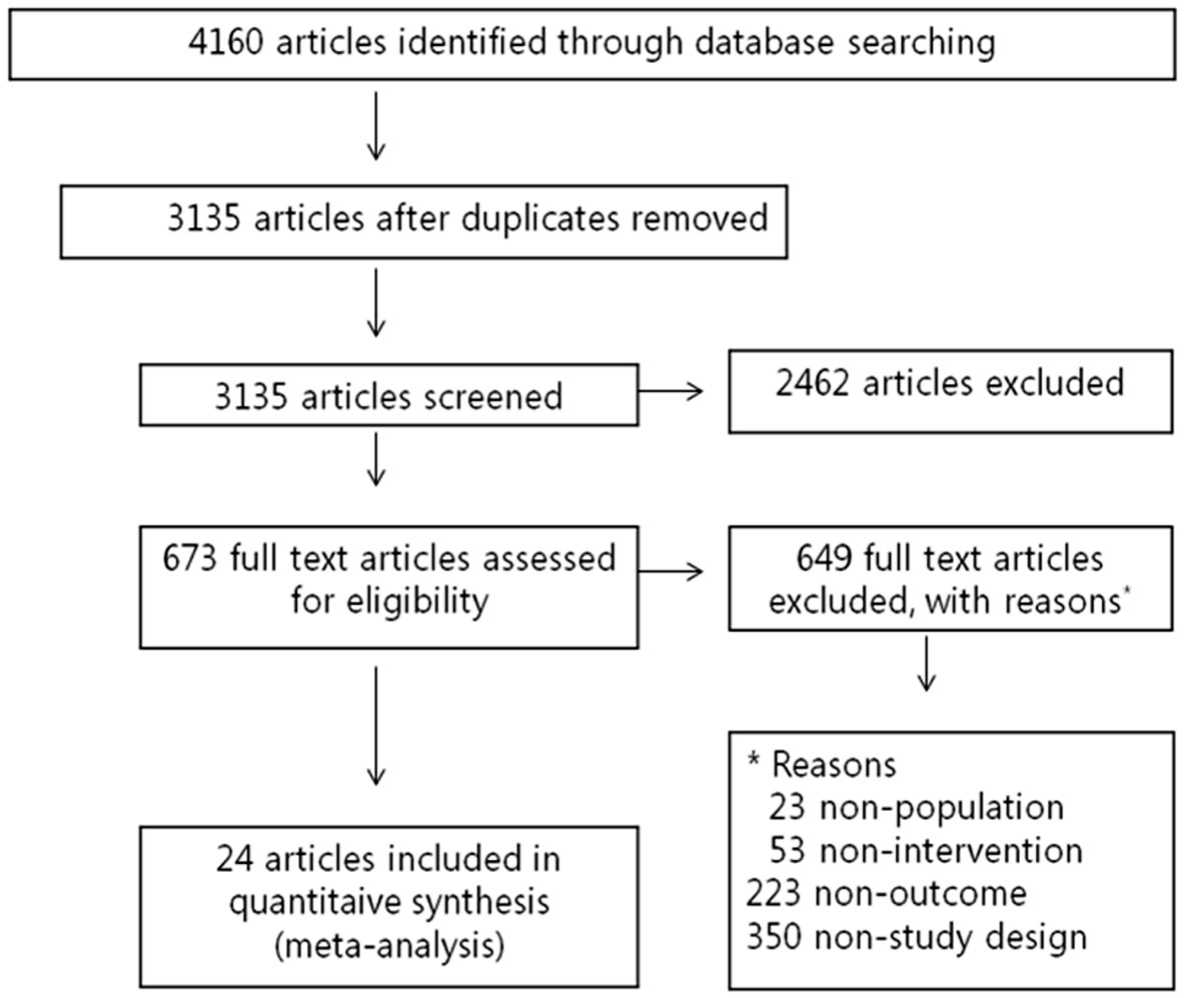
Flow diagram of article selection for this study

The authors independently reviewed titles and abstracts of 3135 identified unique studies; 2462 studies were excluded. The full-text publications of the remaining 673 potentially eligible studies were reviewed in detail. Of these, 645 were excluded based on the inclusion and exclusion criteria (Figure 1). Of 1581 international studies, 1480 Korean domestic studies and 1099 Japanese domestic studies, 24 observational studies reporting data from 31 865 patients were selected for the comprehensive meta-analysis. Characteristics of these studies are presented in Additional file 1 and the quality of studies are listed in Additional file 1.

### Meta-analysis

**Figure 2:**
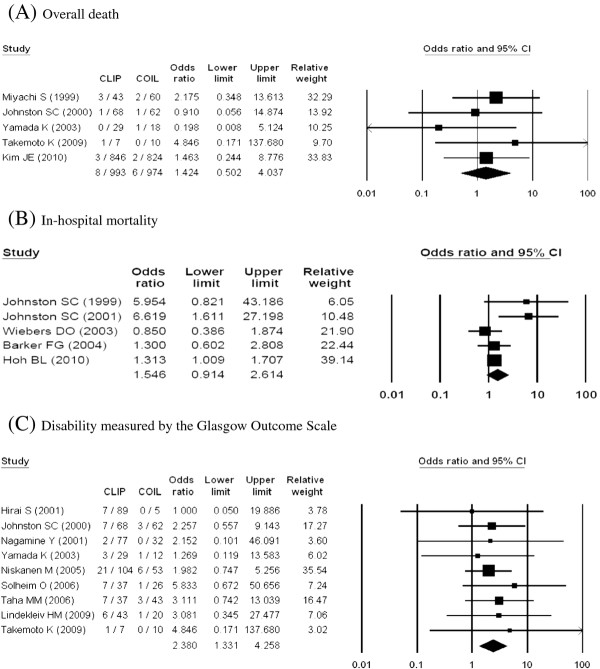
Outcomes from neurosurgical clipping vs. endovascular coiling

**Figure 3:**
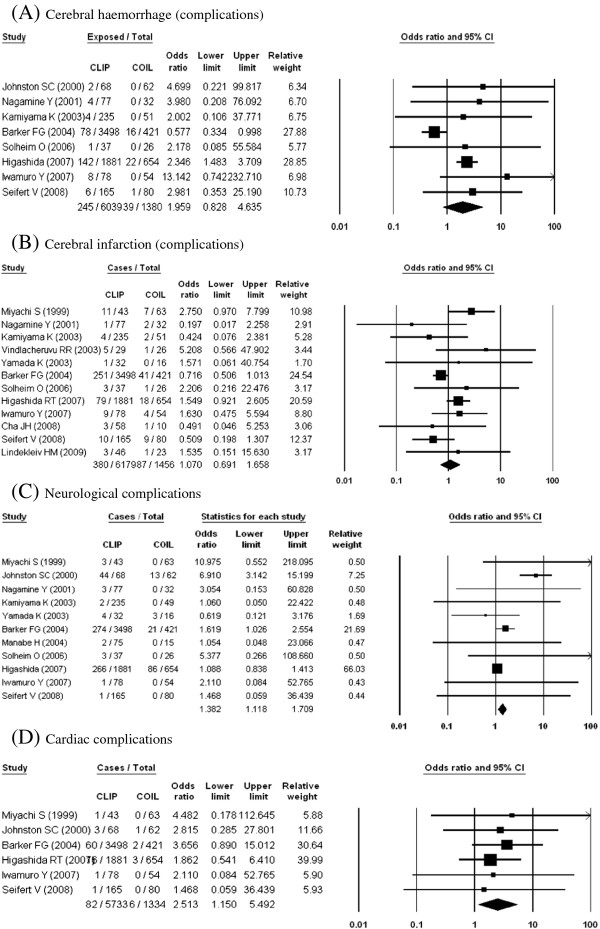
Outcomes from neurosurgical clipping vs. endovascular coiling (complications)

Four outcome types (disability measured by mRS and GOS, neurological and cardiac complications) showed statistically significant differences between coiling and clipping (Table 1, Figure 2, Figure 3). Clipping had significantly higher disability as measured by GOS (OR, 2.38; 95% CI, 1.33–4.26) and mRS (OR, 2.83; 95% CI, 1.42–5.63) when compared with coiling. ORs for neurological (OR, 1.94; 95% CI, 1.09–3.47) and cardiac (OR, 2.51; 95% CI, 1.15–5.50) complications were also higher with clipping.

**Table 1:** Meta-analysis of neurosurgical clipping vs. endovascular coiling for each outcome

**Outcome**	**Studies,*n***	**Patients,*n***	**Random effects, OR (95% CI)**	**Fixed effects, OR (95% CI)**	**I**^**2**^,%*	HeterogeneityP*
Overall death						
Overall	5	1967	1.42 (0.50, 4.04)	1.42 (0.50, 4.04)	0	0.69
≤6 months measurement time	4	1950	1.25 (0.42, 3.74)	1.25 (0.42, 3.74)	0	0.65
In-hospital mortality						
Overall	5		1.55 (0.91, 2.61)	1.35 (1.07, 1.70)	52	0.08
Adjusted OR	3		3.35 (0.91, 12.30)	3.46 (2.20, 5.44)	83	0.003
Disability (GOS)						
Overall	9	754	2.38 (1.33, 4.26)	2.38 (1.33, 4.26)	0	0.99
≤6 months measurement time	4	314	2.72 (1.16, 6.34)	2.72 (1.16, 6.34)	0	0.81
>6 months measurement time	4	331	2.12 (0.93, 4.84)	2.12 (0.93, 4.84)	0	0.90
Asian races	4	413	2.41 (1.19, 4.90)	2.41 (1.19, 4.90)	0	0.84
Western races	5	341	2.32 (0.84, 6.39)	2.32 (0.84, 6.39)	0	0.92
Disability (mRS)	2	262	2.83 (1.42, 5.63)	2.83 (1.42, 5.63)	0	0.81
					0	0.72
Complication						
Cerebral haemorrhage	8	7419	1.96 (0.83, 4.64)	1.44 (1.03, 2.02)	63	0.01
Cerebral infarction	12	7635	1.07 (0.69, 1.66)	0.96 (0.74, 1,23)	39	0.08
Neurological complication	11	7661	1.94 (1.09-3.47)	1.38 (1.12, 1.71)	58	0.009
Cardiac complication	6	7067	2.51 (1.15. 5.50)	2.51 (1.15. 5.50)	0	0.98

Abbreviations: *OR*, odds ratio; *CI*, confidence interval; *mRS*, modified Rankin Scale; *GOS*, Glasgow Outcome Scale; *Values for a random-effects model are reported.

On the other hand, overall mortality, in-hospital mortality and complications of cerebral haemorrhage and cerebral infarction showed no statistically significant differences between the two groups. There was evidence of statistical heterogeneity, and the I^2^ range was 39% to 83%, which represents moderate or substantial heterogeneity. Therefore, overall mortality, in-hospital mortality and complications of cerebral haemorrhage and cerebral infarction were incorporated into a random-effects model.

In subgroup analysis by outcome-measurement time, clipping showed significantly higher disability measured by GOS (OR, 2.72; 95% CI, 1.16–6.34) in the short term (≤6 m). However, disability (GOS) was not significantly different in the long term (>6 m) (OR, 2.12; 95% CI, 0.93–4.84) (Table 1).

We found no apparent systematic bias in the overall and subgroup meta-analyses, as assessed by a funnel plot and test (Table 2).

**Table 2:** Publication-bias test

**Outcome**	**Egger Test *P*-value**	**Begg Test *P*-value**
Overall death	0.5762	1.000
In-hospital mortality	0.3218	0.4624
Disability (GOS)	0.7071	0.9170
Complication		
Cerebral haemorrhage	0.4102	0.9015
Cerebral infarction	0.5302	0.6312
Neurological complication	0.2501	0.8763
Cardiac complication	0.8927	0.7071

### Additional analysis

The re-intervention rate for endovascular coiling was 2% to 15.4% in five studies. Coil compaction within the follow-up period is a common cause in most cases. In contrast, only two clipping studies reported re-intervention; the rate of neurosurgical clipping was 0% in one study and 2.3% in the other.

## Discussion

This study showed that coiling is associated with less harm than clipping in terms of disability measured by mRS and GOS, neurological and cardiac complications, but all of the studies included were observational. It is difficult to randomise controlled trials of surgical intervention in terms of such factors; consequently, observational studies may be the best available evidence. This is the first comprehensive systematic review and meta-analysis comparing clipping and coiling in patients with UIA. King et al. [11] and Raaymakers et al. [12] found mortality rates of 1.0% and 2.6% and morbidity rates of 4.1% and 10.9%, respectively, in meta-analyses of the outcome of clipping for UIA. Ontario [13] performed a systematic review on the outcome of clipping for intracranial aneurysms including UIA. These previous studies were systematic reviews of only a single intervention and did not compare interventions with one another. Therefore, the present study is meaningful despite some limitations.

Sixteen accessible electronic databases were searched, including major international databases and Korean and Japanese domestic databases without language restriction. The aneurismal subarachnoid haemorrhage incidence rate in Japan is more than double those in other regions [14], so Japanese domestic databases were included in the search.

After an extensive search and review, 24 studies were identified (*n*  =  31 865) among 4160 studies. Clipping resulted in significantly higher disability as measured by GOS (overall and short-term [≤6 m]), but this significant difference between the two groups disappeared over the long term (>6 m). Clipping is considered a type of craniotomy and a more invasive treatment than coiling. Therefore, patients may suffer short-term and/or long-term disability as a result of surgery. However, some of these disabilities may disappear over time with healing and therapy.

Clipping had 2–2.5 times as many neurological complications and cardiac complications than coiling. The major complication differed based on the treatment method; for example, ischemic injury caused by vasoocclusion and vasoconstriction during surgery was common in clipping, whereas thromboembolism caused by the endovascular procedure and procedure-related rupture during endovascular therapy were common in coiling.

However, although the cause of ischemic or haemorrhagic brain injury differed, the types of injury were similar (ischemic or haemorrhagic). Therefore, these complications were directly compared between the clipping and coiling groups. No significant differences were found between clipping and coiling in the present study. However, another study using the national impatient sample database showed that in-hospital deaths and perioperative complications (i.e., intracerebral haemorrhage and acute ischemic stroke) were higher with clipping than with coiling [15].

This study had several limitations. First, the selected studies were all observational, because we did not find any well-designed, prospective, randomised comparative clinical trials. Therefore, the levels of evidence for all outcomes were “very low” in keeping with the quality of evidence assessment dictated by GRADEprofiler. Second, the analysis did not examine outcomes according to the size and location of aneurysms. The information about size/location of aneurysms in the selected studies was either not found or was useless because treatment outcomes were not reported for each size and location.

## Conclusions

In conclusion, endovascular coiling has benefits in UIA treatment in terms of low disability after treatment in the short term and fewer complications compared with neurosurgical clipping. However, because most of the reviewed studies were retrospective cohort studies, further well-designed, prospective, randomised comparative clinical trials are required to verify these results.

## Competing interests

The author(s) declare that we have no competing interests.

## Authors' contributions

MKH, Draft the protocol. M K H, JEC, Develop a search strategy. MKH, JEC, Search for articles. NRL, EJL, Obtain copies of articles. HJL, JEC, JSH,MKH, Select which articles to include. HJL, JEC, JSH,MKH, Extract data from articles. HJL, JEC, JHK, JSH, NRL, MKH, Evaluate the quality of articles. MKH, JSH, Carry out the analysis. MKH, JSH, Interpret the analysis. MKH, JSH, JWK, Draft the final review. MKH, Update the review. All authors read and approved the final manuscript.

## Pre-publication history

The pre-publication history for this paper can be accessed here:

http://www.biomedcentral.com/1471-2377/12/99/prepub

## Supplementary Material

Additional file 1 Characteristics of identified publications. Click here for file
