# Supplementary material for: Endovascular coiling versus neurosurgical clipping in patients with unruptured intracranial aneurysm: a systematic review
Source: BMC Neurol. 2012 Sep 22;12:99. doi: 10.1186/1471-2377-12-99 (PMC3519507; doi:10.1186/1471-2377-12-99)
Supplement: Additional file 1 — Characteristics of identified publications. [file 1471-2377-12-99-S1.doc]

**Appendix 1. Characteristics of identified publications**

| **Study** | **Study location** | | **Database** | **Outcome** | **Outcome**  **measurement time** | **No. of patients** | | **Enroll period** |
| --- | --- | --- | --- | --- | --- | --- | --- | --- |
|  |  |  |  |  |  | **Clipping** | **Coiling** |  |
| Johnston SC  (1999) | US | | University Health System Consortium database: 60 university hospitals | In-hospital death | Discharge | 2357 | 255 | 1994-1997 |
| Miyachi S  (1999) | Japan | | Nagoya University School of Medicine | Overall death  Cerebral infarction  Neurological complication  Cardiac complication | 3 months | 43 | 60 | 1990-1998 |
| Johnston SC  (2000) | US | | University of California at San Francisco (UCSF) | Overall death  Modified Rankin Scale  Glasgow Outcome Scale  Intracerebral bleeding  Neurological complication  Cardiac complication | Discharge | 68 | 62 | 1990 |
| Hirai S (2001) | Japan | | Chiba Cardiovascular Center | Glasgow Outcome Scale | - | 89 | 5 | 1982-1999 |
| Johnston SC  (2001) | US | | The Office of Statewide Health Planning and Development of California | In-hospital death  ~~Length of stay~~ | Discharge | 1699 | 370 | 1990-1998 |
| Nagamine Y  (2001) | Japan | | Kohnan Hospital | Glasgow Outcome Scale  Intracerebral bleeding  Cerebral infarction  Neurological complication | - | 75 | 32 | 1995-1998 |
| Kamiyama K  (2003) | Japan | | Nakamura Memorial Hospital | Intracerebral bleeding  Cerebral infarction  Neurological complication | - | 219 | 49 | 1997.6-2001.8 |
| Vindlacheruvu RR (2003) | England | | Middlesbrough General Hospital | Cerebral infarction | 6 months | 65 | 38 | 1996.1-1999.12 |
| Wiebers DO  (2003) | US + Europe | | International Study of Unruptured Intracranial Aneurysms Centre | In-hospital death | 1 month / 1 year | 1917 | 451 | 1991.12-1998.12 |
| Yamada K  (2003) | Japan | | Kyoto University Graduate School of Medicine | Overall death  Glasgow Outcome Scale  Cerebral infarction  Neurological complication | - | 29 | 18 | 1986.10-2002.7 |
| Barker FG  (2004) | US | | Nationwide Inpatient Sample Database | In-hospital death  Intracerebral bleeding  Cerebral infarction  Neurological complication  Cardiac complication | Discharge | 3498 | 421 | 1996-2000 |
| Manabe H  (2004) | Japan | | Kuroishi City Hospital | Neurological complication | - | 75 | 15 | 1996.4-2002.4 |
| Niskanen M  (2005) | Finland | | Kuopio University Hospital | In-hospital death  Glasgow Outcome Scale | 12 months | 105 | 53 | 1997-2000 |
| Solheim O (2006) | Norway | | St. Olav University Hospital | Glasgow Outcome Scale  Intracerebral bleeding  Cerebral infarction  Neurological complication | 6-12 weeks | 37 | 26 | 2000.1-2005.7 |
| Taha MM (2006) | Japan | | Kokura Memorial Hospital | Glasgow Outcome Scale | 3 months | 37 | 43 | 2001.10-2004.10 |
| Cowan JA (2007) | US | | National Inpatient Sample Database | In-hospital death | Discharge | 4715 | 3619 | 1998-2003 |
| Higashida RT  (2007) | | US | 429 hospitals in 18 states  (California, Colorado, Florida, Illinois, Iowa, Maryland, Massachusetts, Nevada, New Hampshire, New Jersey, New York, Oregon, Pennsylvania, Texas, Utah, Vermont, Virginia, Wisconsin) | In-hospital death  Intracerebral bleeding  Cerebral infarction  Neurological complication  Cardiac complication | - | 1881 | 654 | 1998-2000 |
| Iwamuro Y  (2007) | | Japan | Kokura Memorial Hospital | Modified Rankin Scale  Intracerebral bleeding  Cerebral infarction  Neurological complication  Cardiac complication | 3 months | 78 | 54 | 2000-2005 |
| Cha JH  (2008) | | Korea | Busan Catholic Health System | Cerebral infarction | - | 58 | 10 | 1990-2005 |
| Seifert V  (2008) | | Germany | University of Frankfurt | Intracerebral bleeding  Cerebral infarction  Neurological complication  Cardiac complication | 6 months | 126 | 74 | 1999.6-2007.2 |
| Hoh BL  (2010) | | US | Nationwide Inpatient Sample Database | In-hospital death  ~~Length of stay~~ | - | 4513 | 4661 | 2002-2006 |
| Lindekleiv HM  (2009) | | Norway | University Hospital of North Norway | Glasgow Outcome Scale  Cerebral infarction | 12 months | 46 | 23 | 2000-2006 |
| Takemoto K  (2009) | | Japan | Fukuoka University School of Medicine plus  Kyushu Medical Center | Overall death  Glasgow Outcome Scale | - | 7 | 10 | 1996-2006 |
| Kim JE  (2010) | | Korea | 48 hospitals  (41 university hospitals) | Overall death | - | 75 | 32 | 1995-1998 |

**Appendix 2. The quality of studies using the Methodological Index for Nonrandomised Studies**

| **Study** | **A clearly stated aim** | **Inclusion of consecutive patients** | **Prospective collection of data** | **Endpoints appropriate to the aim of the study** | **Unbiased assessment of the study endpoint** | **Follow-up period appropriate to the aim of the study** | **Loss to follow up less than 5%** | **Prospective calculation of the study size** | **An adequate control group** | **Contemporary groups** | **Baseline equivalence of groups** | **Adequate statistical analyses** |
| --- | --- | --- | --- | --- | --- | --- | --- | --- | --- | --- | --- | --- |
| Johnston SC  (1999) | 2 | 0 | 1 | 2 | 0 | 1 | 0 | 0 | 2 | 2 | 2 | 2 |
| Miyachi S  (1999) | 1 | 1 | 0 | 2 | 0 | 1 | 0 | 0 | 2 | 2 | 0 | 0 |
| Johnston SC  (2000) | 1 | 1 | 0 | 2 | 1 | 2 | 2 | 0 | 2 | 2 | 0 | 2 |
| Hirai S (2001) | 1 | 1 | 1 | 2 | 0 | 2 | 0 | 0 | 2 | 2 | 0 | 0 |
| Johnston SC  (2001) | 2 | 2 | 1 | 2 | 0 | 0 | 0 | 0 | 2 | 2 | 1 | 2 |
| Nagamine Y  (2001) | 1 | 1 | 0 | 1 | 1 | 0 | 0 | 0 | 2 | 2 | 1 | 0 |
| Kamiyama K  (2003) | 1 | 1 | 0 | 1 | 0 | 0 | 0 | 0 | 2 | 2 | 0 | 1 |
| Vindlacheruvu RR (2003) | 2 | 2 | 1 | 1 | 0 | 2 | 0 | 0 | 2 | 2 | 0 | 1 |
| Wiebers DO  (2003) | 2 | 2 | 2 | 2 | 2 | 2 | 2 | 0 | 2 | 2 | 1 | 1 |
| Yamada K  (2003) | 1 | 1 | 0 | 2 | 1 | 0 | 0 | 0 | 2 | 2 | 0 | 0 |
| Barker FG  (2004) | 2 | 2 | 1 | 2 | 0 | 0 | 0 | 0 | 2 | 2 | 2 | 2 |
| Manabe H  (2004) | 1 | 1 | 1 | 2 | 2 | 0 | 0 | 0 | 2 | 2 | 0 | 1 |
| Niskanen M  (2005) | 1 | 1 | 1 | 2 | 0 | 2 | 0 | 0 | 2 | 2 | 1 | 1 |
| Solheim O (2006) | 2 | 1 | 1 | 2 | 0 | 2 | 1 | 0 | 2 | 2 | 2 | 1 |
| Taha MM (2006) | 2 | 2 | 1 | 2 | 0 | 2 | 0 | 0 | 0 | 2 | 0 | 0 |
| Cowan JA (2007) | 1 | 2 | 1 | 2 | 0 | 0 | 0 | 0 | 2 | 2 | 1 | 2 |
| Higashida RT  (2007) | 2 | 1 | 1 | 2 | 0 | 0 | 0 | 0 | 2 | 2 | 1 | 2 |
| Iwamuro Y  (2007) | 2 | 0 | 0 | 2 | 0 | 0 | 0 | 0 | 2 | 2 | 0 | 0 |
| Cha JH  (2008) | 2 | 2 | 1 | 0 | 0 | 0 | 0 | 0 | 2 | 2 | 0 | 0 |
| Seifert V  (2008) | 2 | 2 | 1 | 2 | 0 | 1 | 0 | 0 | 2 | 2 | 1 | 0 |
| Hoh BL  (2010) | 2 | 1 | 0 | 2 | 0 | 0 | 0 | 0 | 2 | 2 | 1 | 2 |
| Lindekleiv HM  (2009) | 2 | 2 | 1 | 2 | 0 | 2 | 1 | 0 | 2 | 2 | 0 | 1 |
| Takemoto K  (2009) | 1 | 2 | 0 | 2 | 2 | 2 | 0 | 0 | 2 | 2 | 0 | 1 |
| Kim JE  (2010) | 2 | 1 | 1 | 2 | 0 | 1 | 0 | 0 | 2 | 2 | 0 | 2 |

Note. The items are scored 0 (not reported), 1 (reported but inadequate) or 2 (reported and adequate)
